# The Physiology of Conception

**Published:** 1894-05

**Authors:** A. D. Barr

**Affiliations:** Calamine, Ark.


### THE PHYSIOLOGY OF CONCEPTION.

By DR. A. D. BARR, Calamine, Ark.

THAT the union of the spermatozoa with the female element is necessary for conception is settled beyond controversy; but concerning the manner of their union nothing has heretofore been known. Without entering into a discussion of the generally accepted theories of conception, I will proceed to give what I believe to be the true explanation of it. The spermatozoa do not enter the ovum at all. The ovum is the center that collects the cells that are to compose the embryo.

The true embryonic cells are secreted by the uterine glands, and, like all other cells, are composed of protoplasm, and are found by microscopical examination in the secretion of the uterine glands of all animals, and in the egg of the fowl.

When sexual intercourse takes place, the uterine glands are stimulated, and pour out their secretion in considerable quantity, and thus prepare for the reception of the fertilizing agent.

The spermatozoa immediately after entering the uterine cavity begin to enter the cells that are secreted by the uterine glands. After the spermatozoa enter the cells, the cells begin to divide, and within twenty-four hours they are greatly divided. The cells formed by the division of the original one are much smaller—about the size of white blood-cells. The spermatozoon, being a living agent, penetrates the cell wall; the head and body soon become absorbed by the cell; the tail is absorbed by the cell wall. Thus the spermatozoon disappears, and its life or motion is changed into the molecular motion of the cell. If an ovum is present in the uterus when the embryonic cells are impregnated, or reach it before they perish, the cells now being endowed with life principle, or rather the kinetic energy of the spermatozoon is converted into the potential energy of the cells, and the ovum acts as the center around which they are organized into a living being; and the life of the new being is the potential energy of the cells again converted into kinetic, or the remanifestation of the life of the spermatozoon.

The idea that the body is produced entirely from the ovum is erroneous. The laws governing the organization of the embryo are analogous to those governing the solar system; the ovum corresponds to the centripetal force, and the embryonic cells to the centrifugal. The result of these two opposing forces on living cells are: the centrifugal force of the cell tends to unlimited division to such a degree as to produce complete destruction of the cell, and the centripetal force of the ovum tends to draw the cells all to itself and thus prevent division; thus the two forces balance each other very nearly. It is the variation of these two forces that results in the differentiation of the cells that compose the different organs of the body. Of course, each individual cell possesses centripetal force; but when the spermatozoon enters the cell and its life is converted into motion, the centripetal force of the cell is completely overcome, and if no other force were present the cell, as before said, would be destroyed by its own force. The blood cells are formed from these same impregnated and divided cells. The blood cells are formed in the cells, or rather the cell is changed into the blood corpuscle. Under the microscope the cell can be seen in different stages of transformation into red blood corpuscles, and are readily distinguishable by their change of color and later by change of shape. I do not think the entire cell is converted into the red blood corpuscle, but is formed from the nucleus of the cell; and during its formation the part that is not necessary for its production is dissolved, and, perhaps, serves some unknown use. In support of the view I have advanced, I will give my own observation, supported by physiological facts already known.

The uterine glands, as before stated, secrete a perfect cell; and if this uterine secretion be examined microscopically a short time after sexual intercourse, the spermatozoa will be seen in great abundance mingled promiscuously among the cells. If the same fluid be examined again after remaining six hours in the uterus, the number of spermatozoa will be greatly lessened, and the older cell will be seen to be undergoing typical cell division. If intercourse takes place and there is very scant secretion from the uterus, the seminal fluid remains in the uterus unchanged, and on microscopical examination it will be seen that the spermatozoa still retain their vitality; of course, there will be found a few embryonic cells which have been entered by spermatozoa and are undergoing or have undergone division.

Under such circumstances the cells are gradually produced, and are entered by the spermatozoa as soon as secreted. If the uterine secretion is present in a normal amount when intercourse occurs, and some of the contents of the uterus, after a period of twenty-four hours, be placed under the microscope, scarcely a spermatozoon will be seen. If some of the contents be taken after remaining in the uterus a short time after intercourse, the spermatozoa can be seen entering the cells, and some will be seen after they have entered, and will be recognized by the motion in the center of the cell.

The same phenomenon occurs in the neuroblastic ovum as in the ova of the vertebrate, with this difference: in the former the true ovum is surrounded by the true embryonic cell before impregnation takes place; while in the latter the embryonic cells are gathered together by the ovum after they are impregnated. That the blastodermic membrane is formed from cells that surround the ovum, and not from cells formed from a division of the ovum after impregnation, I have demonstrated by isolating the hen from the male, and I have always found the ovum surrounded by the embryonic cells wheu unimpregnated, the same as when pregnant, thus showing that pregnancy does not cause the almost unlimited division of a cell all from the entrance of a single spermatozoon. This is manifestly the reason why there is such a great number of spermatozoa. The reason that pregnancy scarcely ever occurs just before or immediately after menstruation, is because in the latter part of the menstrual month those glands are inactive on account of degeneracy, and immediately after menstruation they are not properly formed, and in either case do not secrete the embryonic cells.

The ideas set forth in this paper are the result of a careful study of the subject, extending over a period of eighteen months. The observations were made on the mare, sow, cat and hen. I have also found that the uterine glands of the human female secrete the same cells. I therefore conclude that the embryonic cells are not formed by the division of a pregnant ovum, but are formed separate from the ovum. Each embryonic cell is impregnated by a separate spermatozoon. The ovum is the center that gathers the pregnant embryonic cells together. One spermatozoon is not sufficient to cause pregnancy, but as many as [1] of the embryonic cells are required.

The life of the spermatozoa is transformed into the life or motion of the cells, and is again manifested in the life of the new being.—*St. Louis Medical and Surgical Journal.*

1. The author omits to state the number.

